# The *Disphragis notabilis* (Schaus) species-group in Costa Rica (Lepidoptera, Notodontidae)

**DOI:** 10.3897/zookeys.421.7351

**Published:** 2014-06-27

**Authors:** J. Bolling Sullivan, Michael G. Pogue

**Affiliations:** 1 200 Craven Street, Beaufort, North Carolina 28516; 2 Systematic Entomology Laboratory, PSI, Agricultural Research Service, U. S. Department of Agriculture, c/o Smithsonian Institution, P.O. Box 37012, NMNH, MRC-168, Washington, DC 20013-7012, USA.

**Keywords:** Taxonomy, genitalic variation

## Abstract

The four described taxa in the *Disphragis notabilis* (Schaus) species-group are reviewed, including the types and their dissected genitalia. *Disphragis hemicera* (Schaus), **stat. rev.**, is elevated to species rank, *D. normula* (Dognin) is retained as a synonym of *D. notabilis*, *D. sobolis* Miller is confirmed as distinct from *D. hemicera*, and *D. bifurcata* **sp. n.**, is newly described. Both *D. hemicera* and *D. bifurcata* occur in Costa Rica. The known ranges of the other species are outlined. Defining characters of each species are presented and a key to species is provided. Unusual variation in the genitalia is noted.

## Introduction

The name *Disphragis notabilis* (Schaus), described from French Guiana, has been applied to prominent moths throughout Central and South America. Miller described *Disphragis sobolis* from Ecuador and indicated that genitalic characters reveal yet another member of the complex in Ecuador (Miller and Thiaucourt 2011). Collections of so-called *Disphragis notabilis* from Costa Rica are heterogeneous in maculation and dissections and barcodes reveal two distinct species. *Disphragis notabilis* has two junior synonyms, *Disphragis hemicera* (Schaus) from Costa Rica and *Disphragis normula* (Dognin) from Peru, so it is necessary to examine all named taxa in order to classify the species found in Costa Rica.

## Materials and methods

Photographic methods used herein are described in Sullivan and Adams (2009). Procedures for dissecting and preparing genitalia follow those of Lafontaine (2004) and Pogue and Sullivan (2003). Genitalia are shown as dissected and stained, and flattened or not flattened. DNA sequencing of the barcode fragment of the COI gene was carried out at the Canadian Center for DNA barcoding in Guelph, Ontario. Barcode sequences were compared by nearest neighbor analyses as implemented on the Barcode of Life Data systems website (Ratnasingham and Hebert 2007). Genitalia dissection numbers are given in the format JBS-xxxx, DNA voucher numbers in the format xx-CRBS-xxxx and xx-MISC-xxx.

### Repository abbreviations

BMNH Natural History Museum, London, UK

INBio Instituto Nacional de Biodiversidad, Santo Domingo de Heredia, Costa Rica

JBS J. Bolling Sullivan collection, Beaufort, North Carolina, USA

USNM National Museum of Natural History, Washington, District of Columbia, USA

### Key to species based on male genitalia

**Table:** 

1	Socii short, 1/6 length of valva and upcurved below a short, almost hood-like uncus	2
–	Socii long, ½ length of valva, below triangular “dunce cap” uncus	3
2	Phallus with two plates of flattened spines at base of sclerotized portion	*Disphragis hemicera*
–	Phallus with a parallel extension at base of sclerotized portion	*Disphragis sobolis*
3	Socii large, bifurcated at tip	*Disphragis bifurcata*
–	Socii smaller, tapering to single point with ventral spines	*Disphragis notabilis*

## Systematics

The female type specimen of *Heterocampa notabilis* Schaus was named from French Guiana. Both *Heterocampa hemicera* Schaus (male) from Costa Rica and *Heterocampa normula* Dognin (male) from Peru are listed as synonyms of *Disphragis notabilis* by Gaede (1934). Miller described *Disphragis sobolis* from the mountains of eastern Ecuador and noted that it was easily separated from specimens of *Disphragis notabilis* by its darker color and more mottled appearance (Miller and Thiaucourt 2011). They also illustrate the genitalia of a male of *Disphragis notabilis* from French Guiana and the male genitalia of *Disphragis sobolis*.

### Disphragis bifurcata

Taxon classificationAnimaliaLepidopteraNotodontidae

Sullivan & Pogue sp. n.

http://zoobank.org/0944967F-1CB1-48C6-9702-0B8FA0D8E9B9

**Figures 1–4.**
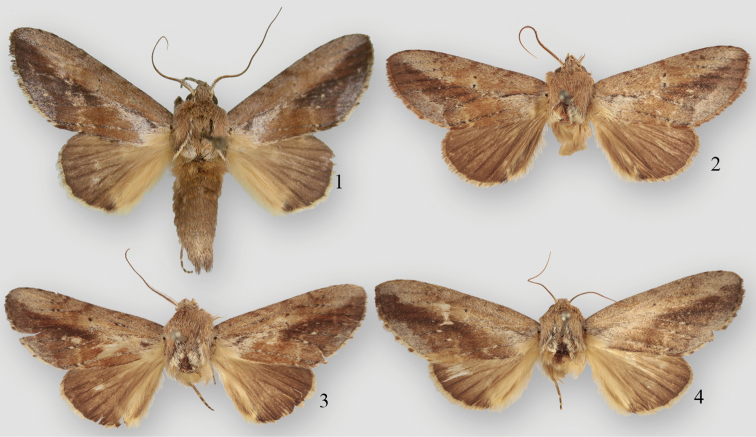
*Disphragis notabilis* complex holotypes. **1** *Disphragis bifurcata*, male holotype **2** *Disphragis hemicera*, male holotype **3** *Disphragis normula*, male holotype **4** *Disphragis notabilis*, female holotype.

Figs 1 , 10 , 14 , 18 , 22

#### Type material.

Holotype male: Costa Rica, Reserva Hitoy Cerere (9.404°N, 83.015°W), Limon Province, 354’, 1–4 July 2008, J. Bolling Sullivan. INBio. **Paratypes:** 11♂, 3♀: 4♂, same data as holotype (JBS-2094, JBS-3053); 1♀, 22 March 2003, Monty Wood (JBS-3030); 1♂, Costa Rica, Est. Biol. La Selva (10.26°N, 84.01°W), Heredia Province, 50–150 m, 21–30 June 2003, Monty Volovsic (JBS-3040), 2♂, 29 Aug. –2 Sept. 2003, J. Bolling Sullivan (JBS-3038); 2♂, Costa Rica, Upata Estacion San Gerardo (10.89°N, 85.38°W), Alajuela Province, 550 m, 17–21 July 2006, J. Bolling Sullivan, B. Espinosa (JBS-3035); 1♂, Costa Rica, Puriscal Chires, Mastatal (N9.411; W-84.220), San Jose Province, 400 m, 16–18 Oct. 2011, J. Bolling Sullivan; 1♂, Costa Rica, Verugua Rainforest Campamento (9.553°N, 83.112°W), Limon Province, 400–500 m, 12–16 March 2010, J. Bolling Sullivan (11-CRBS-2066), (JBS-5427); 1♀, Costa Rica, Tuis, 2500’, June, W. Schaus 1910-110. (BM-); 1♀, Costa Rica, Cashi, 8–10 1912 (Lankester), Rothschild Bequest, B. M. 1939-1. (BM-). (USNM, BMNH, JBS, INBio)

#### Etymology.

The name *bifurcata* refers to the bifurcate tip of the socii, which is diagnostic.

#### Diagnosis.

Maculation characters can usually be used to separate *Disphragis bifurcata* and *Disphragis notabilis* from the other two members of the complex. Forewing color is a warm brown, not mottled or brownish gray as in *Disphragis sobolis* and *Disphragis hemicera*. Additionally, the male antennal pectinations are shorter in *Disphragis bifurcata* and *Disphragis notabilis*. Males of *Disphragis bifurcata* are easily distinguished by the large upturned and bifurcated socii in the male genitalia. In males of *Disphragis notabilis* the socii usually have a single point at the apex, with many spines arising from the ventral edge. Females must be identified by maculation and geography; *Disphragis bifurcata* occurs in Central America and central and western Colombia.

#### Description.

**Male.** (Figs 1, 10) *Head*–labial palps upturned, mahogany brown on basal segment, medial segment with cream scaling along distal margin, particularly near the terminus, and apical segment mostly cream scaled with scattered brown scales. Denuded medial segment 2.4× length of apical segment. Eye round, large, surrounded tightly with scaling. Front scaling mostly cream with scattered brown scales. Vertex with additional brown scales among white scaling. Scape with cream and brown scaling, cream extending onto antennal shaft for about 10–14 segments. Antenna bipectinate basally for 30 segments, then with minute basal seta on segments to tip (68 segments). Longest rami 0.44 mm. Thorax a blend of fine brown and cream scales giving a tan appearance. Metathorax bearing a central white spot with row of darker brown scales anteriorly. Abdomen with appressed brown scaling. Forewing (17.5 mm N = 10) elongate, rounded apically and with broad tan subcostal streak from base of wing to apex. Streak encloses chocolate reniform spot and has several slightly darker brown lines crossing obliquely from costa. Basal dash below streak paralleling costa. White streak below basal dash; warm brown patch distal to white streak bordered by white; wavy antemedial (AM) and postmedial (PM) lines. Chocolate shading from middle of forewing below costal streak and forming a wedge to margin (below costal streak to anal angle). Weak gray crescent on lower half of margin. Hind wing fuscous with darker margin and veins, weak darker brown anal markings almost a spot at anal angle. Underside of forewing fuscous, anal margin and cell yellowish. Basal 3/4 of hind wing yellowish, margin brown and well differentiated. Legs a mixture of brown and white scales, appearing almost yellowish, with white scales forming rings at distal end of tarsal segments. Tibial spines 0-2-4. *Male genitalia* (Figs 14, 18) (8 dissections). Uncus an extended triangle, apex rounded with setae arranged almost in marginal rows. Tegumen broad, longer than vinculum. Socii extending from base of uncus as two large upcurved arms, scythe-like, apex bifurcate. Occasionally tip may be subdivided farther with arrowhead-like plates embedded near apex (visible at higher magnification). However, plates do not form ventral spine-like projections as in *Disphragis notabilis*. Gnathos absent, anal tube membranous. Valva elongated with costal half sclerotized, anal half membranous and enveloping deciduous scent hairs. Valva apex rounded, sclerotized costal half of valva with broad anal projection distally and sharper but rounded and more heavily sclerotized projection basally. Vinculum broad, short, rounded to saccus. Phallus long, narrow with subbasal keel, proximal half unsclerotized, ductus entering medially. Distal half of phallus sclerotized, enlarged basally at junction with membranous half, and with small teeth-like spines ventrally and laterally on basal half. Vesica emerges dorsally from aedeagus, forming a membranous tube that turns to parallel aedeagus and then to left with no major diverticula. A lightly sclerotized sliver-like cornutus often visible and often with small peg-like cornuti where vesica turns left. Eighth tergite broadly rounded, slightly sclerotized and crenulated medially at distal end. Eighth sternite lightly sclerotized, broadly rounded with well-defined, broad notch medially. Small sac-like flap in middle of sclerite, anterior end of sclerite with two broad, rounded projections with medial V-shaped notch. Ctenophores absent. **Female.** (Fig. 10). Female similar to male only larger (Forewing 21.3 mm, *n* = 3) and with fasciculate antennae. *Female genitalia* (Fig. 22) (3 dissections). Papillae anales bluntly rounded, slightly setose. Extension of 9^th^ tergite forming dorsal flap. Anterior apophysis short, 25% as long as posterior apophysis. Genital plate small, elongate, consisting of a bifurcated middle phalanx with lateral “wings” from base. Ductus bursae slightly shorter than corpus bursae, narrow and tending to twist, unsclerotized. Corpus bursae egg shaped, with large signum on dorsal surface. Signum shield-like, about half as long as corpus bursae. Signum egg shaped with stippled lateral flanges anterior to midpoint. Proximal margin lightly sclerotized and faintly stippled.

#### DNA barcode sequence.

Five barcoded specimens exhibit two haplotypes that differ from each other by a maximum of 0.15%. They differ from *Disphragis hemicera* by a minimum of 5.61%, from *Disphragis notabilis* by a minimum of 1.26%, and from *Disphragis sobolis* by a minimum of 5.78%. The most common haplotype (11-CRBS-2066) is:

AACCTTATATTTCATTTTTGGAATTTGAGCAGGAATAGTAGGAACCTCTTTAAGTCTTCTAATTCGTGCTGAATTAGGAACCCCCGGGACTTTAATTGGAGATGATCAAATTTATAATACTATTGTAACAGCTCATGCTTTCATTATAATTTTTTTTATAGTAATACCTATTATAATTGGAGGATTTGGAAATTGATTAGTACCTTTAATATTAGGAGCCCCAGACATAGCTTTCCCACGAATAAATAATATAAGTTTTTGATTATTACCTCCTTCTTTAATACTTTTAATTTCGAGAAGTATTGTAGAAAATGGAGCAGGAACAGGATGAACAGTTTACCCACCACTGTCATCTAATATTGCTCATAGAGGAAGCTCTGTTGATTTAGCCATTTTTTCCCTTCACTTAGCTGGTATTTCATCAATTTTAGGGGCTATTAATTTTATCACAACAATTATTAATATACGATTAAATAATATATCTTTTGATCAAATACCTTTATTTGTGTGAGCTGTAGGAATTACTGCTTTTTTACTTTTACTTTCTCTCCCAGTTCTAGCTGGAGCTATTACTATACTTTTAACTGATCGTAATTTAAATACATCTTTTTTTGACCCTGCAGGGGGAGGAGATCCTATTTTATACCAACATTTATTT

#### Distribution.

Known from Guatemala to Colombia (Anchicaya, Valle, and the Magdalena Valley), and probably extending south into northern Ecuador.

#### Remarks.

This species occurs at lower altitudes and moderate elevations (1000 m) where it occurs with *Disphragis hemicera*.

### Disphragis hemicera

Taxon classificationAnimaliaLepidopteraNotodontidae

(Schaus, 1910) stat. rev.

Figs 2 , 5 , 11 , 15 , 19 , 23 , 26–29

Heterocampa hemicera Schaus, 1910, Annals and Magazine of Natural History 6: 582.

#### Type locality.

Costa Rica.

#### Diagnosis.

Maculation will usually separate *Disphragis hemicera* and *Disphragis sobolis* from *Disphragis notabilis* and *Disphragis bifurcata*. Their appearance is mottled, grayish brown with a distinct dark band next to the PM line. Males may be distinguished by the shape of the phallus, which in *Disphragis sobolis* has a distinct dorsal projection. Females can be separated by the shape of the genital plate, which in *Disphragis sobolis* is bifurcate at the distal tip and in *Disphragis hemicera* has a middle phalanx with lateral “wings” from the base. Geographic distribution also separates *Disphragis hemicera* from *Disphragis sobolis*, with *Disphragis hemicera* in Central America and western Colombia, and *Disphragis sobolis* along the western slopes of the Andes.

#### Description.

**Figures 5–9.**
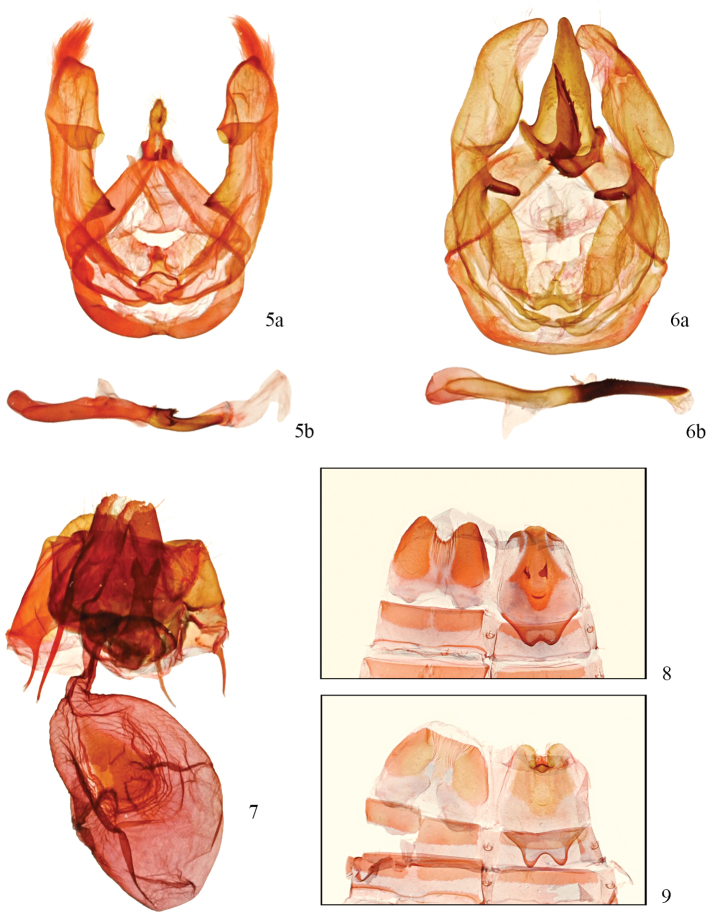
*Disphragis notabilis* complex holotype genitalia. **5** *Disphragis hemicera*, male holotype (USNM-49851) **a** valve **b** phallus **6** *Disphragis normula*, male holotype (USNM-49852) **a** valve **b** phallus **7** *Disphragis notabilis*, female holotype (USNM-49853) **8** *Disphragis hemicera* male holotype (USNM-49851) tergites **9** *Disphragis normula* male holotype (USNM-49852) tergites.

**Male.** (Figs 2, 5) *Head*–labial palp upturned, mahogany brown on basal segment, medial segment with cream scaling along distal margin particularly near the terminus, and apical segment mostly cream scaled with scattered brown scales. Denuded medial segment is 3.2× length of apical segment that is shortened relative to *Disphragis bifurcata*. Eye round, large, surrounded tightly with scaling. Front scaling mostly cream with scattered brown scales. Vertex with additional brown scales among cream scaling. Scape with cream and brown scaling, cream scaling extending onto antennal shaft for about 14–18 segments. Antenna bipectinated basally for 33 segments, then with minute basal seta on segments to apex (73 segments). Rami noticeably longer than in *Disphragis bifurcata*, longest rami 0.53 mm. Thorax a blend of brown and cream scales giving a tan appearance. Metathorax with a central white spot with row of darker brown scales anteriorly. Abdomen with appressed brown scaling. Forewing (17.9 mm, n = 10) elongate, rounded apically and with broad light brown subcostal streak from base of wing to apex. Streak encloses chocolate reniform spot and has several slightly darker brown lines crossing obliquely from costa. Basal dash below streak perpendicular to thorax. White streak below dash; warm brown patch distal to white streak bordered by white; AM and PM lines wavy. Distinct brown line bisecting warm brown patch. Chocolate shading from middle of forewing below costal streak and forming a wedge to margin (below costal streak to anal angle). Gray crescent on lower half of margin with distinct brown band inward to PM line. Hind wing uniformly fuscous with brown anal markings forming something of a spot at anal margin. Light streak along anal edge. Underside of forewing fuscous with yellowish subapical crescent along costa. Basal half of hind wing yellowish, no well-differentiated margin. Legs a mixture of brown and white scales appearing somewhat yellowish with white scales forming rings at distal end of tarsal joints. Tibial spines 0-2-4. *Male genitalia* (Figs 15, 19) (12 dissections). Uncus lightly sclerotized and rounded, turning 90 degrees ventrally and forming a rounded, setose pad. Socii small, upturned and pointed slightly, blade-like. Tegumen broad, triangular, similar in size to vinculum. Valve elongated rounded at apex and costal half sclerotized. Anal half of valve membranous and enclosing deciduous hair-like scent scales. Distal third of valve enlarged dorsally ending abruptly with shelf-like narrowing. Second narrowing of sclerotized subcostal area 1/3 distance from base, a rounded projection less shelf-like that distal projection, but more heavily sclerotized. Juxta shovel shaped with handle toward aedeagus. Vinculum rounded to saccus. Aedeagus long, narrow and with basal 2/3 membranous, subbasal keel present. Distal 1/3 sclerotized with two prominent toothed plates at junction with membranous portion. Vesica tube-like emerging dorsally then turning 90 degrees forward to plane of phallus. Distinct lateral diverticulum to left of midpoint. Cornuti absent. Ctenophore on pelt absent. Eighth tergite broadly rounded, slightly sclerotized and crenulated medially at distal end. Sternite lightly sclerotized with “happy face” consisting of two membranous flaps for “eyes” and a broad anterior one for “mouth.” Anterior edge tapered to blunt, indented terminus. **Female.** (Fig. 5). Female similar to male only larger (Forewing 21.0 mm, n = 5) and with fasciculate antennae. *Female genitalia* (Figs 23, 26–29) (10 dissections). Papillae anales bluntly rounded, slightly setose. Extension of 9^th^ tergite forming dorsal flap in *Disphragis bifurcata* greatly reduced to small crescent in *Disphragis hemicera*. Anterior apophysis short, 25% as long as posterior apophysis. Genital plate small, slightly elongate, consisting of a middle phalanx with lateral “wings” from base. Phalanx usually shorter than in *Disphragis bifurcata*. Tip of phalanx variable, usually blunt but can be indented or bifurcate. Ductus bursae slightly shorter than corpus bursae, narrow and tending to twist, membraneous. Corpus bursae egg shaped with large signum on dorsal surface. Signum shield-like, about half as long as corpus bursae. Signum egg shaped with stippled lateral flanges below midpoint. Proximal margin lightly sclerotized and faintly stippled.

**Figures 10–13.**
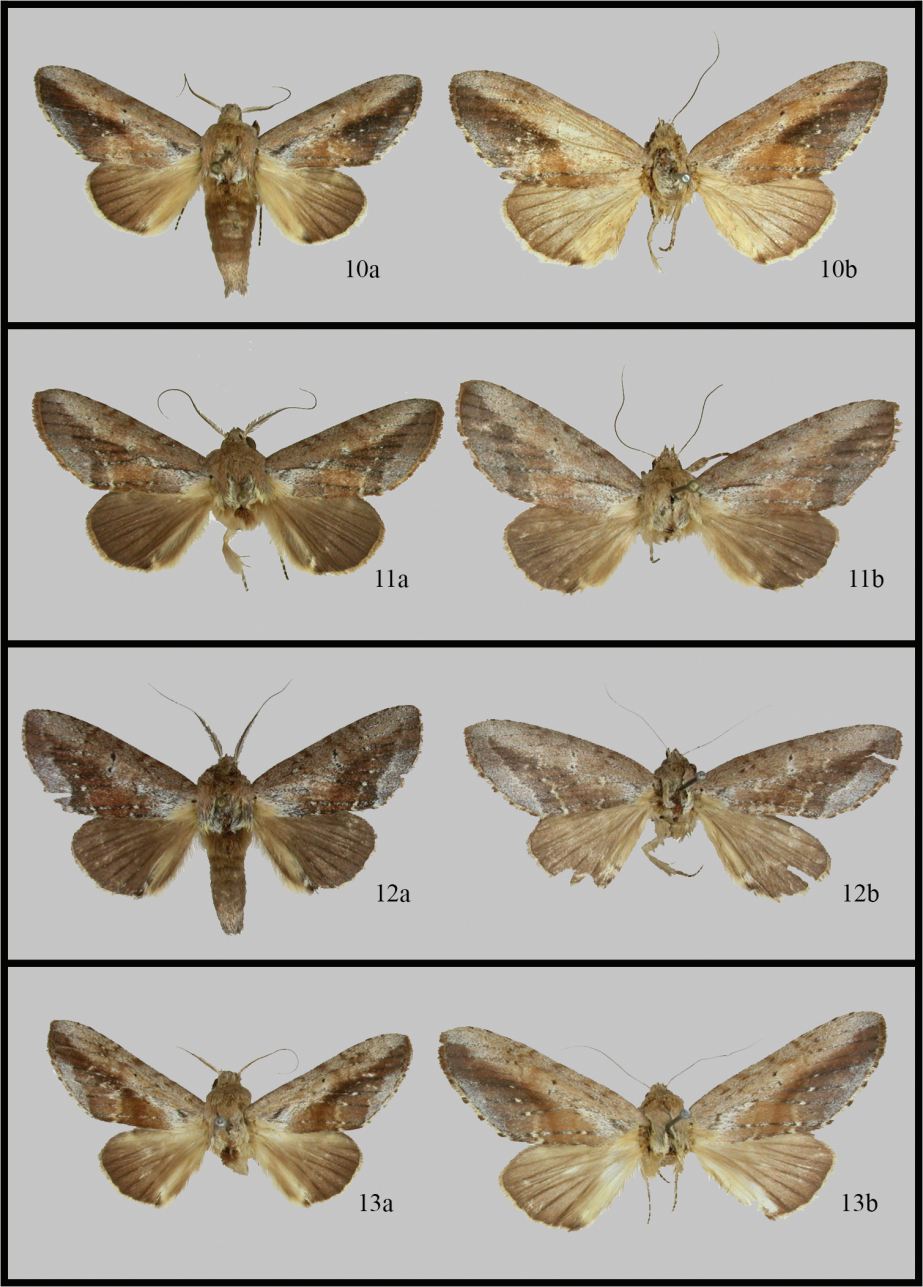
*Disphragis notabilis* complex adults. **10** *Disphragis bifurcata* **a** male (Costa Rica) **b** female (Costa Rica) **11** *Disphragis hemicera* **a** male (Costa Rica) **b** female (Costa Rica) **12** *Disphragis sobolis* **a** male (Ecuador) **b** female (Peru) **13** *Disphragis notabilis* **a** male (French Guiana **b** female (Brazil).

**Figures 14–16.**
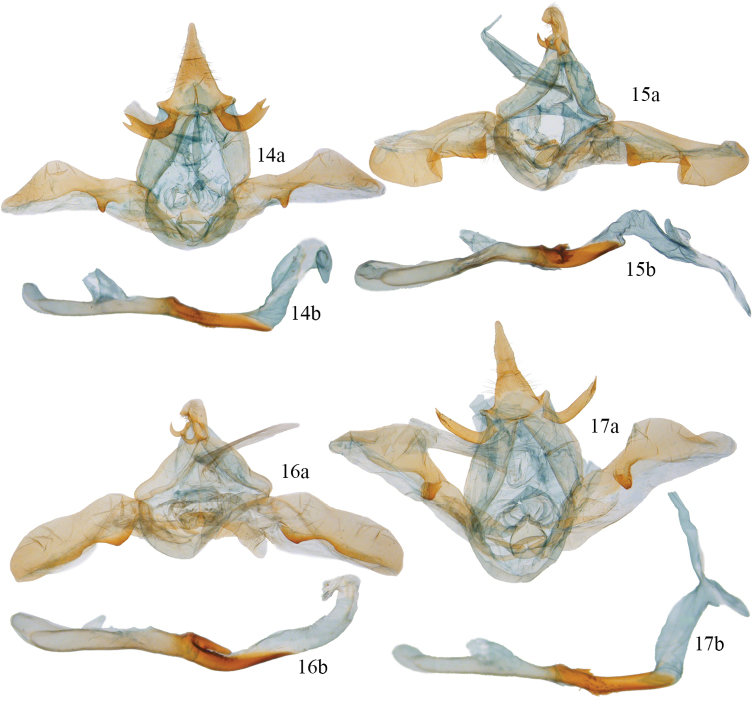
*Disphragis notabilis* complex male valves and phalli. **14** *Disphragis bifurcata* (JBS-3035) **a** valve **b** phallus **15** *Disphragis hemicera* (JBS-3037) **a** valve **b** phallus **16** *Disphragis sobolis* (BMNH-NOTO1964) **a** valve **b** phallus **17** *Disphragis notabilis* (BMNH-NOTO1968) **a** valve **b** phallus.

**Figures 18–21.**
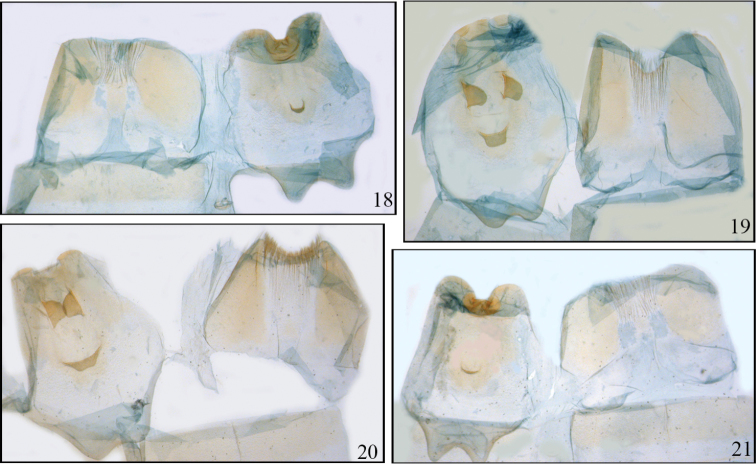
*Disphragis notabilis* complex male tergites. **18** *Disphragis bifurcata* (JBS-3035) **19** *Disphragis hemicera* (JBS-3037) **20** *Disphragis sobolis* (BMNH-NOTO1964) **21** *Disphragis notabilis* (BMNH-NOTO1968).

**Figures 22–25.**
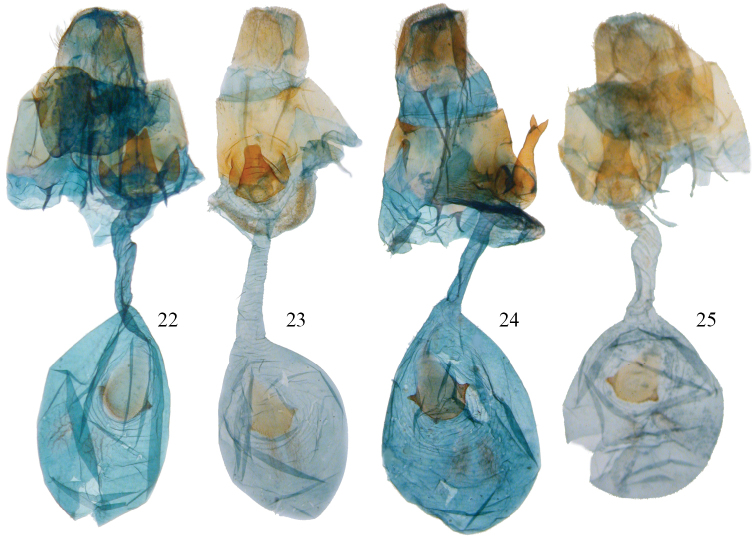
*Disphragis notabilis* complex female genitalia. **22** *Disphragis bifurcata* (BMNH-NOTO1984) **23** *Disphragis hemicera* (JBS-3049) **24** *Disphragis sobolis* (BMNH-NOTO1988) **25** *Disphragis notabilis* (BMNH-NOTO1972).

**Figure 26–29.**
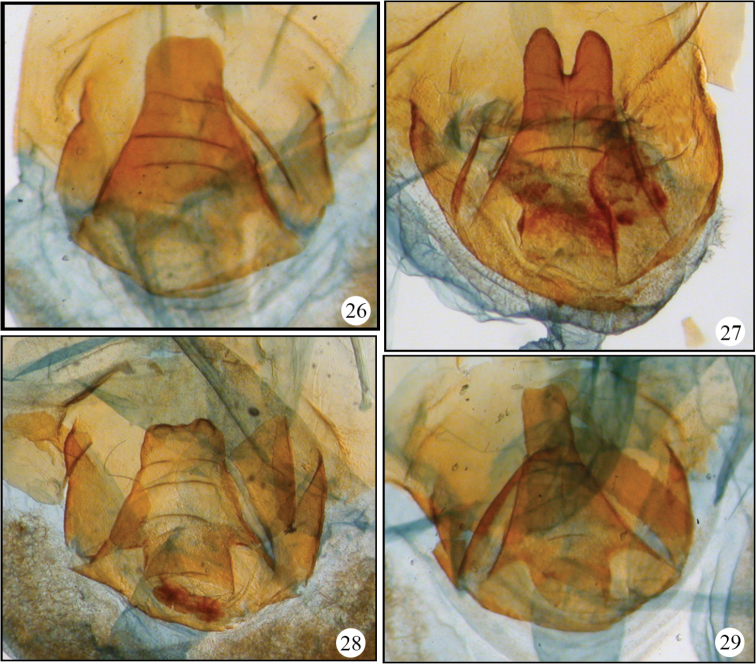
Variable genital plates of *Disphratis hemicera* from Costa Rica. **26** Costa Rica (JBS-3049) **27** Costa Rica (JBS-3041) **28** Costa Rica (JBS-3050) **29** Costa Rica (JBS-3046).

#### Barcodes.

Fifty eight barcoded specimens exhibit seven haplotypes that differ from each other by a maximum of 0.30%. They differ from those of *Disphragis bifurcata* by a minimum of 5.61%, from *Disphragis notabilis* by a minimum of 5.65%, and from *Disphragis sobolis* by a minimum of 6.13%. The most common haplotype (11-CRBS-2519) is:

AACTTTATATTTTATTTTTGGAATTTGAGCAGGAATAGTAGGAACTTCTTTAAGTCTTTTAATTCGTGCTGAATTAGGAACCCCCGGGACTTTAATTGGAGATGATCAAATTTATAATACTATCGTAACAGCTCATGCTTTTATTATAATTTTTTTTATAGTTATACCTATTATAATTGGAGGATTTGGAAATTGATTAGTCCCTTTAATACTAGGAGCACCAGATATAGCTTTCCCACGAATAAATAATATAAGTTTTTGACTATTACCCCCTTCTTTAATACTTCTAATTTCAAGAAGTATTGTAGAAAATGGAGCTGGTACAGGATGAACAGTTTATCCCCCACTGTCATCAAATATTGCTCACGGAGGAAGCTCTGTTGATTTAGCTATTTTTTCCCTTCATTTAGCGGGTATTTCCTCAATTTTAGGGGCTATTAATTTTATTACAACAATTATTAATATACGATTAAATAATATATCTTTTGATCAAATACCTTTATTTGTATGAGCTGTAGGAATTACTGCTTTTCTACTTTTACTTTCACTCCCAGTATTAGCTGGAGCTATTACTATACTTTTAACCGATCGTAATTTAAATACATCTTTTTTCGACCCTGCTGGGGGAGGAGATCCTATTTTATACCAACATTTATTT

#### Distribution.

*Disphragis hemicera* occurs throughout Costa Rica at moderate altitudes. It is found south along the western coast of Colombia and may extend to the west coast of Ecuador. The northern limits are unknown but it probably occurs at least into Nicaragua.

#### Remarks.

*Disphragis hemicera* is by far the most common member of the group in Costa Rica and appears to be absent below 500 m. At moderate altitudes both *Disphragis hemicera* and *Disphragis bifurcata* occur together.

### Disphragis notabilis

Taxon classificationAnimaliaLepidopteraNotodontidae

(Schaus, 1906)

Figs 4 , 7 , 13 , 17 , 21 , 25

Heterocampa notabilis Schaus, 1906, Proceedings of the United States National Museum 29: 253.
Heterocampa normula Dognin, 1909, Annales de la Société entomologique de Belgique 53: 81. (Fig. 3)

#### Type locality.

*Disphragis notabilis*: French Guiana; *Disphragis normula*: Peru

#### Diagnosis.

Maculation characters can usually be used to separate *Disphragis notabilis* and *Disphragis bifurcata* from *Disphragis hemicera* and *Disphragis sobolis*. *Disphragis notabilis* and *Disphragis bifurcata* are warm brown, not mottled or brownish gray like *Disphragis hemicera* and *Disphragis sobolis*. The male antennal pectinations are shorter in *Disphragis notabilis* than in *Disphragis hemicera* and *Disphragis sobolis*. Males of *Disphragis notabilis* are easily distinguished by their moderately wide socii, which taper to a single point with many ventral spines. In males of *Disphragis bifurcata* the socii are much broader and are bifurcate at the upturned apex. Females must be sorted by maculation and geography. *Disphragis notabilis* is Amazonian in distribution whereas *Disphragis bifurcata* occurs from central and western Colombia north into Central America.

#### Description.

**Male.** (Fig. 13) *Head*–palp upturned, mahogany brown on basal segment, medial segment with cream scaling along distal margin particularly near terminus; apical segment mostly cream with scattered brown scales. Denuded medial segment 4.1× length of apical segment. Apical segment reduced in size relative to other species in complex. Eye round, large, surrounded tightly with scaling. Front scaling mostly cream with scattered brown scales. Vertex with additional brown scales among white scaling. Scape with cream and brown scaling, white scaling extending onto antennal shaft for about 10–14 segments. Antenna bipectinate basally for 29 segments then with minute basal setae on segments to apex (71 segments). Longest rami 0.34 mm, shortest of all species. Thorax a blend of brown and cream scales giving a tan appearance. Metathorax bearing a central white spot with row of darker brown scales anteriorly. Abdomen with appressed brown scaling. Forewing (17.0 mm, n = 10) elongate, rounded apically and with broad tan subcostal streak from base of wing to apex. Streak encloses chocolate reniform spot and has several slightly darker brown lines crossing obliquely from costa. Basal dash below streak perpendicular to thorax, abbreviated relative to that of *Disphragis bifurcata*. White streak below dash; warm brown patch distal to white streak bordered by white; AM and PM lines wavy. Chocolate shading from middle of wing below costal streak and forming a wedge to margin (below costal streak to above mid point of margin). Weak gray crescent on lower half of margin. Warm brown from patch expanded almost to margin and reducing size of chocolate wedge seen in *Disphragis bifurcata*. Hind wing fuscous with darker margin, weak darker brown anal markings almost forming a spot. Underside of forewing fuscous, anal margin and cell yellowish. Basal 3/4 of hind wing yellowish, margin brown and moderately differentiated. Legs a mixture of brown and white scales appearing almost yellowish with white scales forming rings at distal end of tarsal joints. Tibial spines 0-2-4. *Male genitalia* (Figs 17, 21) (13 dissections). Uncus an extended triangle, rounded apex with setae arranged almost in marginal rows. Tegumen broad, longer than vinculum. Socii extending from base of uncus as two upcurved arms, scythe-like with small, spine-like projections on ventral surface. Degree of spination variable from several to many extending down to angle of socius. Gnathos absent, anal tube unsclerotized. Valve elongated with costal half sclerotized, anal half membranous and enveloping deciduous scent hairs. Valve apex rounded, sclerotized costal half of valva with broad anal projection distally and sharper shelf-like projection basally. Vinculum broad, short and rounded to saccus. Aedeagus long, narrow with basal phallus, proximal 60% unsclerotized with ductus entering medially. Distal 40% of aedeagus sclerotized, enlarged basally at junction with membranous half, and with raised mound of spines ventrally about 1/3 distal from junction. Vesica emerges dorsally from aedeagus, an unsclerotized tube with a long dorsal diverticulum. Cornuti absent. Eighth tergite broadly rounded, slightly sclerotized and crenulated medially at distal end. Eighth sternite lightly sclerotized, broadly rounded with well-defined, broad notch medially, usually broader than in *Disphragis bifurcata*. Small sac-like flap in middle of sclerite usually in form of narrow crescent, anterior end of sclerite with two broad, rounded projections with medial V-shaped notch. Ctenophores absent on pelt. **Female.** (Figs 4, 13). Female similar to male only larger (Forewing 20.9 mm, n = 6) and with fasciculate antennae. *Female genitalia* (Fig. 25) (5 dissections). Papillae anales bluntly rounded, slightly setose. Extension of 9^th^ tergite forming dorsal flap. Anterior apophysis short, 25% as long as posterior apophysis. Genital plate small, elongate, consisting of a bifurcated middle phalanx with lateral “wings” from base. Phalanx somewhat longer than in *Disphragis bifurcata*. Ductus bursae slightly shorter than corpus bursae, twice as wide as in *Disphragis bifurcata* and tending to twist, unsclerotized. Corpus bursae egg-shaped with large signum on dorsal side. Signum shield-like, about half as long as corpus bursae. Signum egg shaped with stipulated lateral flanges below midpoint. Proximal margin lightly sclerotized and faintly stippled.

#### Barcodes.

Two barcoded specimens exhibit 2 haplotypes that differ from each other by 0.30%. They differ from those of *Disphragis hemicera* by a minimum of 5.65%, from *Disphragis bifurcata* by a minimum of 1.26%, and from *Disphragis sobolis* by a minimum of 4.78%. One haplotype (11-MISC-302) is:

AACTTTATATTTCATTTTTGGAATTTGAGCAGGAATAGTAGGAACCTCTTTAAGTCTTCTAATTCGTGCTGAATTAGGAACCCCCGGGACTTTAATTGGAGATGACCAAATTTATAATACTATCGTAACAGCTCATGCTTTCATTATAATTTTTTTTATAGTAATACCTATTATAATTGGAGGATTTGGAAATTGATTAGTACCTTTAATATTAGGAGCCCCAGACATAGCTTTCCCACGAATAAATAATATAAGTTTTTGATTATTACCTCCTTCTTTAATACTTTTAATTTCAAGAAGTATTGTAGAAAATGGAGCAGGAACAGGATGAACAGTTTACCCACCACTGTCATCTAATATTGCCCATAGAGGAAGCTCTGTTGATTTAGCCATTTTTTCCCTTCACTTAGCCGGTATTTCATCAATTTTAGGGGCTATTAATTTTATCACAACAATTATTAATATACGATTAAATAATATATCTTTTGATCAAATACCTTTATTTGTATGAGCTGTAGGAATTACTGCTTTTTTACTTTTACTTTCTCTTCCAGTTCTAGCTGGAGCTATTACTATACTTTTAACTGATCGTAATTTAAATACATCTTTTTTTGACCCTGCAGGGGGAGGAGATCCTATTTTATACCAACATTTATTT

#### Distribution.

This species occurs throughout the Amazon basin from western Venezuela eastward and southward to at least Bolivia.

#### Remarks.

*Disphragis notabilis* is by far the most common member of the group in South America, however, earlier references to this species should be confirmed in light of the additional species described here.

### Disphragis sobolis

Taxon classificationAnimaliaLepidopteraNotodontidae

Miller, 2011

Figs 12 , 16 , 20 , 24

Disphragis sobolis Miller, 2011. In Miller and Thiaucourt 2011, Annals of the Entomological Society of America 104: 1058.

#### Type locality.

Ecuador.

#### Description.

**Male.** (Fig. 12) *Head*–labial palpus upturned, mahogany brown on basal segment, medial segment with cream scaling along distal margin, particularly near the terminus, and apical segment mostly cream scaled with scattered brown scales. Denuded medial segment 2.6× length of apical segment. Eye round, large, surrounded tightly with scaling. Front scaling mostly cream with scattered brown scales. Vertex with additional brown scales among cream scaling. Scape with cream and brown scaling, white scaling extending onto antennal shaft for about 14–18 segments. Antenna bipectinated basally for 33 segments then with minute basal seta on segments to tip (68 segments). Rami noticeably longer than in *Disphragis hemicera*, longest 0.59 mm. Thorax a blend of brown and cream scales giving a tan appearance. Metathorax bearing a central white spot with row of darker brown scales anteriorly. Abdomen with appressed brown scaling. Forewing (19.3 mm, n = 5) elongate, rounded apically and with broad light brown subcostal streak from base of wing to apex. Streak encloses chocolate reniform spot and has several slightly darker brown lines crossing obliquely from costa. Brown scaling throughout as well as several black streaks. Basal dash below streak perpendicular to thorax and greatly reduced in length. White streak below dash; warm brown patch distal to white streak bordered by white; AM and PM lines wavy. Distinct brown line bisecting warm brown patch. Chocolate shading from middle of forewing below costal streak and forming a wedge to margin (below costal streak to anal angle) more extensive than in *Disphragis hemicera*. Prominent gray crescent on lower half of margin with distinct brown band inward to PM line. Hind wing uniformly fuscous with brown anal markings almost a spot. Light streak along anal edge. Underside of forewing fuscous with yellowish subapical crescent along costa. Basal half of hind wing yellowish, no well-differentiated margin. Legs a mixture of brown and white scales, appearing almost yellowish with white scales forming rings at distal end of tarsal joints. Tibial spines 0-2-4. *Male genitalia* (Figs 16, 20) (5 dissections). Uncus lightly sclerotized and rounded, turning 90 degrees ventrally and forming a much smaller setose pad than in *Disphragis hemicera*. Socii small but 2× larger than in *Disphragis hemicera*, upturned and pointed slightly, blade-like. Tegumen broad, triangular similar in size to vinculum. Valve elongated, rounded at tip and costal half sclerotized. Anal half of valve membranous and enclosing deciduous hair-like scent scales. Distal third of valve considerably enlarged dorsally then gradually narrowing. Second narrowing of sclerotized subcostal area 1/3 distance from base, a rounded projection, more heavily sclerotized. Juxta shovel shaped with handle toward aedeagus. Vinculum rounded to saccus. Aedeagus long, narrow and with basal 2/3 membranous, aedeagus present. Distal 1/3 sclerotized with prominent basal process. Vesica tube-like emerging dorsally then turning 90° to plane of aedeagus. Distinct lateral diverticulum to left of midpoint. Cornuti absent. Ctenophore absent on pelt. Eighth tergite broadly rounded, slightly sclerotized and crenulated medially at distal end. Eighth sternite lightly sclerotized with “happy face” consisting of two membranous flaps for “eyes” and a broad anterior one for “mouth.” Anterior edge tapers to blunt, indented terminus. **Female.** (Fig. 12) Female similar to male only larger and with fasciculate antennae. *Female genitalia* (Fig. 24) (3 dissections). Papillae anales bluntly rounded, slightly setose. Extension of 9^th^ tergite forming dorsal flap in *Disphragis bifurcata* and *Disphragis notabilis* greatly reduced to small crescent in *Disphragis sobolis*. Anterior apophysis short, 25% as long as posterior apophysis. Genital plate small, elongated, consisting of a middle phalanx with lateral “wings” from base. Phalanx longer than in other related species. Tip of phalanx forming a Y-shape. Ductus bursae slightly shorter than corpus bursae, narrow and tending to twist, unsclerotized. Corpus bursae egg shaped with large signum on dorsal surface. Signum shield-like, about half as long as corpus bursae. Signum egg shaped with stipulated lateral flanges below midpoint. Proximal margin lightly sclerotized and faintly stippled.

#### Barcodes.

One specimen has been barcoded and differs from that of *Disphragis hemicera* by a minimum of 6.13%, from *Disphragis bifurcata* by a minimum of 5.78%, and from *Disphragis notabilis* by a minimum of 4.78%. The haplotype (11-MISC-495) is:

AACTTTATATTTTATTTTTGGAATTTGAGCAGGAATAGTAGGAACCTCTTTAAGTCTCCTAATTCGTGCTGAATTAGGAACCCCCGGGACTTTAATTGGAGATGATCAAATTTATAATACTATTGTAACAGCTCATGCTTTTATTATAATTTTTTTTATAGTAATACCCATTATAATTGGAGGATTTGGTAATTGATTAGTTCCTCTAATATTAGGAGCTCCAGATATAGCTTTCCCACGAATAAATAATATAAGTTTTTGATTATTACCCCCCTCTCTAATACTTTTAATTTCAAGAAGTATTGTAGAAAATGGAGCAGGAACAGGATGAACAGTTTACCCCCCACTGTCATCAAACATTGCTCATAGAGGAAGATCTGTTGATTTAGCTATTTTTTCCCTTCACTTAGCAGGTATTTCATCAATTTTAGGAGCTATTAATTTTATTACAACAATTATTAATATACGATTAAATAACATATCTTTTGATCAAATACCTTTATTTGTTTGAGCTGTAGGAATTACTGCTTTTTTACTTTTACTCTCTCTTCCAGTATTAGCAGGAGCTATTACTATATTATTAACCGATCGTAATTTAAATACATCTTTTTTTGACCCCGCTGGGGGAGGAGATCCTATTTTATATCAACATTTATTT

#### Distribution.

This species appears to be limited to the eastern slopes of the Andes from Bolivia to Villavicencio, Colombia.

#### Remarks.

*Disphragis sobolis* was recently described from Ecuador; the species appears to have a much greater geographical range and occurs to almost 3000 m. The lower altitude limits of its range are undefined, as is the southern boundary.

## Discussion

The *Disphragis notabilis* complex is typical of many neotropical species. When studied in detail, they frequently are found to consist of a number of very similar species that do have structural differences, can be separated by barcoding, and occupy different altitudes or geographic ranges. The correct generic placement of this complex is not in *Disphragis* (as placed by Schintlmeister 2013), but its phylogenetic relationships remain unresolved. In general, we find most neotropical notodontid genera to be heterogeneous. The structures of the unci and socii in the *Disphragis notabilis* complex are divergent and illustrate the difficulty in proper generic identification. Although all 4 species are very similar in maculation, they fall into two distinct groupings based on the shape of the uncus and socii. In the *Disphragis notabilis* group, the uncus is large, triangular and elongate. In the *Disphragis hemicera* group the uncus is short, robust and bent ventrally. Normally, differences of this magnitude would warrant separate genera but the slight differences in maculation and female genitalia are quite normal for closely related species. If such dramatic transitions are common among the notodontids, correctly associating species into monophyletic genera will continue to be difficult.

A second difficulty encountered in this study was the hyper-variation of the female genital plate in *Disphragis hemicera* (Figs 26–29). This intraspecific variation (in individuals with identical barcodes) has also been seen in *Didugua* Druce (JBS, unpubl. data) and makes the delineation of species and their defining characters with female specimens extremely difficult. Fortunately, such extreme variation has not been observed in males.

The exact distribution of the four species of the *Disphragis notabilis* complex found in Colombia (and probably Ecuador) remains to be elucidated but should highlight the individual habitat requirements of each species. Neither larvae nor foodplants are known. The geographical area where *Disphragis bifurcata* and *Disphragis notabilis* come into contact should be particularly interesting because the two species differ by 1.4% in their barcodes, a magnitude lower than between most congeneric species. However, there are a number of characters that separate the two species and characters do not seem to intergrade in individuals from central Colombia and western Venezuela.

## Supplementary Material

XML Treatment for Disphragis bifurcata

XML Treatment for Disphragis hemicera

XML Treatment for Disphragis notabilis

XML Treatment for Disphragis sobolis
